# Nongenetic and Genetic Factors Associated with White Matter Brain Aging: Exposome-Wide and Genome-Wide Association Study

**DOI:** 10.3390/genes15101285

**Published:** 2024-09-30

**Authors:** Li Feng, Halley S. Milleson, Zhenyao Ye, Travis Canida, Hongjie Ke, Menglu Liang, Si Gao, Shuo Chen, L. Elliot Hong, Peter Kochunov, David K. Y. Lei, Tianzhou Ma

**Affiliations:** 1 Department of Nutrition and Food Science, College of Agriculture & Natural Resources, University of Maryland, College Park, MD 20740, USA; fenglicindy91314@gmail.com (L.F.); dlei@umd.edu (D.K.Y.L.); 2 Department of Epidemiology and Biostatistics, School of Public Health, University of Maryland, College Park, MD 20740, USA; hmilleso@umd.edu (H.S.M.); tacanida@umd.edu (T.C.); kehj@terpmail.umd.edu (H.K.); mliang23@umd.edu (M.L.); 3 Department of Mathematics, The College of Computer, Mathematical, and Natural Sciences, University of Maryland, College Park, MD 20740, USA; 4 Maryland Psychiatric Research Center, Department of Psychiatry, School of Medicine, University of Maryland, Baltimore, MD 21228, USA; zye@som.umaryland.edu (Z.Y.); si.gao@uth.tmc.edu (S.G.); shuochen@som.umaryland.edu (S.C.); 5 Division of Biostatistics and Bioinformatics, Department of Epidemiology and Public Health, School of Medicine, University of Maryland, Baltimore, MD 21201, USA; 6 Louis A. Faillace Department of Psychiatry & Behavioral Sciences, McGovern Medical School, University of Texas Health Science Center at Houston, Houston, TX 77030, USA; l.elliot.hong@uth.tmc.edu (L.E.H.); peter.kochunov@uth.tmc.edu (P.K.)

**Keywords:** white matter brain aging, XWAS, GWAS, UK Biobank

## Abstract

Background/Objectives: Human brain aging is a complex process that affects various aspects of brain function and structure, increasing susceptibility to neurological and psychiatric disorders. A number of nongenetic (e.g., environmental and lifestyle) and genetic risk factors are found to contribute to the varying rates at which the brain ages among individuals. Methods: In this paper, we conducted both an exposome-wide association study (XWAS) and a genome-wide association study (GWAS) on white matter brain aging in the UK Biobank, revealing the multifactorial nature of brain aging. We applied a machine learning algorithm and leveraged fractional anisotropy tract measurements from diffusion tensor imaging data to predict the white matter brain age gap (BAG) and treated it as the marker of brain aging. For XWAS, we included 107 variables encompassing five major categories of modifiable exposures that potentially impact brain aging and performed both univariate and multivariate analysis to select the final set of nongenetic risk factors. Results: We found current tobacco smoking, dietary habits including oily fish, beef, lamb, cereal, and coffee intake, length of mobile phone use, use of UV protection, and frequency of solarium/sunlamp use were associated with the BAG. In genetic analysis, we identified several SNPs on chromosome 3 mapped to genes IP6K1, GMNC, OSTN, and SLC25A20 significantly associated with the BAG, showing the high heritability and polygenic architecture of human brain aging. Conclusions: The critical nongenetic and genetic risk factors identified in our study provide insights into the causal relationship between white matter brain aging and neurodegenerative diseases.

## 1. Introduction

Brain aging involves the gradual loss of structure and function of neurons and their connections, leading to cognitive decline and increased vulnerability to neurodegenerative diseases [1,2], which vary significantly among individuals. A number of genetic, environmental, and lifestyle factors were found to be associated with brain aging and to contribute to individual heterogeneity in brain aging [3]. Studying how these factors influence brain aging is critical in understanding the intricate mechanisms behind cognitive decline and the onset of neurodegenerative diseases. Numerous studies have identified individual risk factors linked to brain aging, such as chronic smoking [4,5,6], poor dietary habits [7,8,9,10], high blood pressure [11,12], and a high allostatic load [13], which negatively impact brain health. Excessive electronic device use, particularly digital use, has also been suggested to impair cognitive functions [1,2]. While previous research has explored many of these factors in isolation, some recent studies have adopted a broader framework for understanding brain aging. For example, the tripartite model of the exposome highlights the complex interactions between environmental exposures, genetics, and brain connectivity [14]. This model emphasizes the need to account for the interconnected nature of exposures and their genetic modifiers. Additionally, several studies have also leveraged the UK Biobank dataset to explore brain age prediction and associations with various factors, including lifestyle, cognitive, and biomedical variables [15,16,17], and gene–environment interaction [18,19]. These studies have contributed significantly to our understanding of brain aging, yet the complexity of multiple exposures and their potential interactions remains underexplored. To address this gap, we conducted an exposome-wide association study (XWAS) of brain aging by systematically evaluating a wide range of (modifiable) exposure variables encompassing five major categories: electronic device use, smoking, diet, alcohol consumption, and sun exposure. This comprehensive approach allowed for the analysis of both individual exposures and their potential combined effects on brain aging. Unlike traditional hypothesis-driven methods, which may lead to biased estimates or overlook interactions, XWAS identifies complex relationships between multiple exposures and brain aging. Having adopted this holistic approach, our findings offer a deeper understanding of the multifactorial nature of brain aging and provide crucial insights for developing public health strategies to mitigate cognitive decline at earlier stages.

We employed a machine learning algorithm [20,21], leveraging multiple fractional anisotropy (FA) tract measurements obtained from diffusion tensor imaging (DTI) data, to predict the white matter (WM) brain age gap (BAG) as a marker of brain aging [12]. DTI is an advanced magnetic resonance imaging (MRI) technique that measures the diffusion of water molecules along white matter tracts, providing insights into white matter integrity. Water diffusion is directionally restricted in healthy white matter, and FA values, which range from 0 (completely random diffusion, indicating damaged or less structured tissue) to 1 (highly directional diffusion, reflecting intact white matter), quantify this anisotropy [22,23,24]. BAG represents the difference between an individual’s chronological and biological brain age. Increases in BAG imply accelerated aging and poorer physical and brain health [11,12,25,26,27]. The scaler BAG is a robust, easily interpretable metric that simplifies complex multivariate analyses [20,21,28], making it ideal for XWAS analysis of brain aging.

The BAG is highly heritable [29], but the genetic underpinnings of the BAG are not completely understood. In addition to examining multiple environmental and lifestyle factors in XWAS, we also assessed the genetic susceptibility to the BAG by conducting a genome-wide association study followed by using a polygenic risk score (PRS) analysis [30,31,32]. Further, we also explored the potential interactions between the PRS and individual environmental exposures, to study whether individuals with a higher genetic risk are more susceptible to the detrimental effects of specific environmental factors. This gene–environment interaction approach can help identify high-risk subgroups and inform targeted intervention strategies.

In this study, we performed both an XWAS and a GWAS on the BAG and evaluated the effects of a wide range of nongenetic and genetic risk factors on brain aging in the large-scale population-based UK Biobank cohort. This comprehensive analysis aimed to highlight the complex and multifaceted nature of brain aging, emphasizing the need for a holistic approach to identify genetic and modifiable risk factors and develop targeted interventions for healthier aging.

## 2. Methods

### 2.1. Study Population

**Figure 1:**
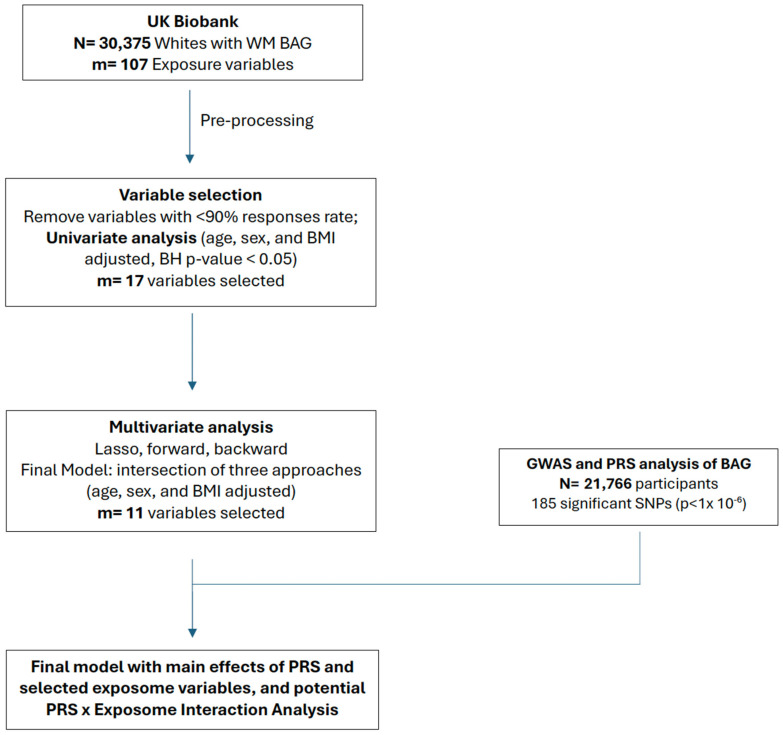
Overview of the analytical steps in this study.

The data used in this study were extracted from the UK Biobank (UKB), a large-scale prospective cohort with deep genetic, environmental, lifestyle, and health data collected from roughly 500,000 individuals in the UK aged 40–69 at recruitment during 2006–2010. Beginning in 2014, the brain imaging data (N~40 k) were collected [33], based on which we derived our outcome of interest, the white matter brain age gap (WM BAG). Following our previous studies, here, we focused on non-pregnant and white (self-reported) participants in the UKB to avoid imbalanced bias and reduce heterogeneity across populations in training the brain age prediction model [11,12]. The participants who had fractional anisotropy (FA) and exposure data available but did not have extreme WM hyperintensities (N = 30,375) were used for our exposome-wide association analysis (Figure 1).

### 2.2. Exposure Variables

In this study, we conducted an XWAS using 107 relevant environmental and lifestyle variables from self-reported questionnaire data, which fell into five categories: electronic device use (11 variables), smoking (33 variables), diet (33 variables), alcohol (21 variables), and sun exposure (9 variables). All variables were carefully preprocessed, re-coded, and transformed when necessary. For ease of interpretation and avoidance of overfitting, ordinal categorical variables (e.g., beef and oily fish intake in the diet category) were treated as numerical, assuming the numerical distance between two neighboring categories was the same. For nominal categorical variables (e.g., plays computer games in the electronic device use category), we followed binary coding [34], where we treated the most frequent level as the reference level and merged the remaining levels as the other level. Variables with an <90% response rate were removed to ensure the data quality. For the remaining variables, we considered an available case analysis in the univariate analysis stage and complete case analysis in the multivariate analysis stage.

### 2.3. White Matter Brain Age Gap (WM BAG)

The detailed method for WM brain age gap (BAG) estimation can be found in our previous studies [12,13,27]. In brief, we calculated the BAG using FA values from DTI data in the UK Biobank (Instance 2, 2014+) [12]. The diffusion-weighted MRI (dMRI) data comprise two shells with b-values of 1000 and 2000 s/mm^2^, each containing 50 distinct diffusion-encoding directions, and with 5 b = 0 images (FOV = 104 × 104 × 72, duration = 7 min). We focused on the DTI FA images in a 2 × 2 × 2 mm space, which were processed using tract-skeleton processing (e.g., tract-based spatial statistics (TBSS) analysis) [35]. The FA images were aligned onto a standard-space white matter skeleton, and the skeletonized images were averaged within each standard-space tract mask, as defined by Susumu Mori’s group at Johns Hopkins University [36,37]. The UKB acquisition protocol adheres to the ENIGMA structural and DTI pipelines [38]. Full details of the image preprocessing and analysis are available in the UKB (biobank.ctsu.ox.ac.uk/crystal/crystal/docs/brain_mri.pdf (accessed on 1 July 2024)). Briefly, the UKB workflow provides measurements evaluated across ENGIMA studies [39], encompassing 39 regional WM tract FA values. The TBSS analysis was conducted at a voxel level, with region-wise metrics obtained by averaging intra-ROI voxel measures [38]. Additionally, the UKB used an automated quality control tool to identify usable and non-usable images, aiming to minimize the false negative rate without significantly increasing the false positive rate [40]. Therefore, FA measurements were derived from 39 white matter tracts covering various brain regions [35,41]. A random forest regression model was trained with FA data to predict brain age, using healthy participants in the training set (N = 8570), excluding those with conditions like smoking, hypertension, and comorbidities [20,21]. From the 39 FA regions, the model selected 19 FA tracts that were most predictive of brain age (Table S1). Hyperparameters of the random forest model included 200 decision trees, a maximum tree depth of 10, and the number of features considered at each split set to the square root of the total number of features. The model used a minimum of 5 samples required to split an internal node, and a minimum of 2 samples at each leaf node. Additionally, bootstrapping was applied to ensure robustness. The model was optimized using 5-fold cross-validation and then applied to a testing set (N = 30,375) to predict brain age. The WM BAG was obtained by subtracting chronological age from the predicted brain age, with bias correction performed using linear regression to reduce age-related bias (Figure S1) [42,43].

### 2.4. Genotype Data

In addition to XWAS on the BAG, we also conducted a genome-wide association study (GWAS) on the BAG followed by polygenic risk score (PRS) analysis to investigate the genetic risk of the BAG and its potential interaction with identified environmental and lifestyle variables.

The UKB genotype data were generated for over 90 million single-nucleotide variants from approximately 500,000 subjects using two microarray platforms. We applied quality control using PLINK (version 1.9) [44] based on the following criteria: single-nucleotide polymorphisms (SNPs) with a 95% genotyping rate, a minor allele frequency ≥ 0.01, and passing the Hardy–Weinberg test at 0.001. Individuals with more than 2% missing genotypes were excluded. A total of N = 21,766 participants who had FA data and passed the filtering criteria remained for the GWAS analysis (Figure 1).

### 2.5. Statistical Analysis

#### 2.5.1. Exposome-Wide Association Study (XWAS)

We first performed univariate linear regression analysis of the BAG on each exposure variable and kept only significant variables (BH adjusted *p* < 0.05) for the next stage, multivariate analysis. Next, for the multivariate analysis, we ran several model selection approaches (Lasso, forward and backward selection) and took the intersection of three approaches as our selection for the final model for robustness (Figure 1).

#### 2.5.2. Genome-Wide Association Studies (GWASs) and Polygenic Risk Score (PRS) Analysis

We conducted a GWAS analysis on the BAG, adjusting for the age at an imaging visit, sex, and genotyping chip type. Using the GWAS summary statistics, we then calculated the PRS to estimate individuals’ genetic risk of the BAG by aggregating the genetic effects from the GWAS. The PRS was generated using unduplicated and non-ambiguous SNPs with a linkage disequilibrium r^2^ ≤ 0.25 at a set of GWAS *p*-value thresholds, e.g., *P_T_* = $1\times 10^{-6},\;1\times 10^{-5},\ldots ,\;0.05,\;0.1,\;\ldots ,\;0.5$. We further calculated an R^2^ of the PRS for distinct *p*-value thresholds by fitting a full model that regresses the BAG on age, sex, genotyping chip type, and PRS, and we compared it to a null model without PRS. Furthermore, we used ANNOVAR [45] to annotate SNPs with GWAS *p*-values < 1 × 10^−6^.

## 3. Results

### 3.1. Descriptive Statistics

The optimal random forest regression model selected 16 fractional anisotropy measures to predict brain age. The age-adjusted predicted brain age achieved a Pearson correlation coefficient (R) = 0.89, and a mean absolute error (MAE) = 2.74 years in the testing dataset (Figure S1), implying an excellent prediction performance of the proposed machine learning model. In the analytical sample of 30,375 participants, the mean WM BAG was 0.13 (SD, 3.45). This consisted of 14,704 men and 15,671 women with a mean age of 55.47 (SD = 7.44). Almost half of the individuals (44.39%) within this study had a college/university degree, and the Townsend deprivation index (mean = −1.86, SD = 2.73) indicating the social deprivation of the group was on the slight negative side (Table 1).

### 3.2. XWAS Analysis

**Figure 2:**
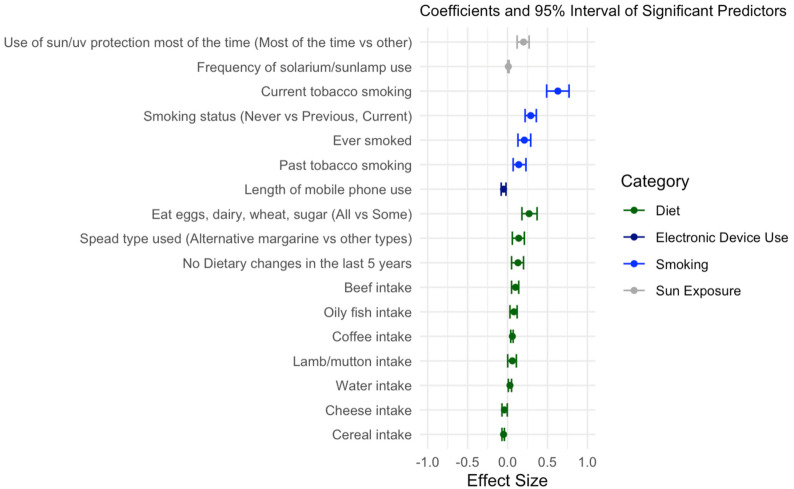
Coefficients and 95% CIs of the 17 exposure variables selected in the univariate analysis of XWAS. Variables are ordered by categories. Dots represents coefficients, and lines represent the 95% CIs.

We regressed the WM BAG on each of the exposure variables, adjusting for the covariates age, sex, and BMI. Upon analysis, a total of 17 variables were found to be significantly associated with the WM BAG (BH adjusted *p*-value < 0.05; see Table 2). These variables fell into four categories: electronic device use, smoking, diet, and sun exposure. Four variables in the smoking category (every smoked, smoking status, current tobacco smoking, and past tobacco smoking) were associated with accelerated brain aging (positive coefficients, see Table 2 and Figure 2), where the current tobacco smoking status had the largest effect size. Under the diet category, the oily fish intake, beef intake, lamb/mutton intake, consumption of eggs/dairy, spread type used, water intake, and dietary changes in the last 5 years were associated with accelerated brain aging, while the cereal and coffee intake were associated with decelerated brain aging, with the majority of these variables having relatively small effect sizes (Figure 2). Length of mobile use was weakly associated with decelerated brain aging, whereas both use of sun/UV protection and frequency of solarium/sunlamp use were associated with accelerated brain aging (Figure 2).

Considering the high correlations among the exposure variables, we further conducted multivariate analysis and performed model selection using Lasso and forward/backward selection. The final model selected 11 exposure variables (Table S2): length of mobile phone use, current tobacco smoking, oily fish intake, beef intake, lamb/mutton intake, cereal intake, coffee intake, water intake, dietary changes in the last 5 years, use of sun/UV protection, and frequency of solarium/sunlamp use. The effect sizes for most of these variables were relatively small. A more sophisticated study design is needed to further investigate the potentially causal relationship between these variables and white matter brain aging.

### 3.3. GWAS and PRS Analysis

**Figure 3:**
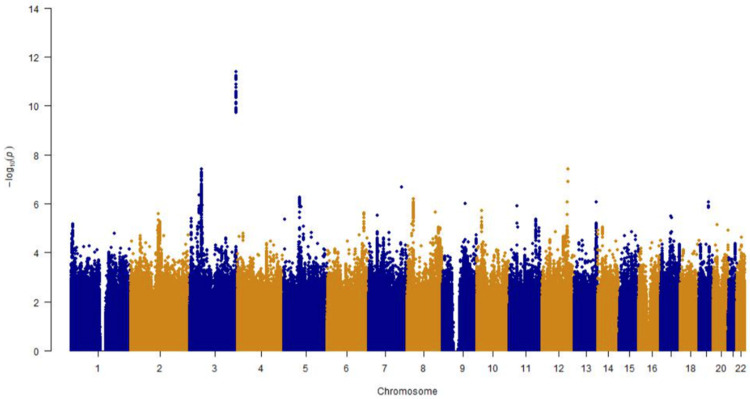
Manhattan plot of association results from GWAS on BAGs. Chromosome numbers are shown on the *x*-axis, and −log10 association *p*-values on the *y*-axis.

The GWAS analysis identified 185 significant GWAS signals (*p* < 1 × 10^−6^) on chromosomes 3 and 5 associated with the BAG, which were mapped to 43 genes. Figure 3 shows the Manhattan plots of the GWAS results, and Table S3 provides an annotation for the 185 significant signals. Among the set of mapped genes, gene *IP6K1* encoding inositol hexakisphosphate kinase has been reported as essential to brain aging in individuals with attention-deficit/hyperactivity disorder, particularly playing a key role in the function of brain insula [46]. Additionally, our top-leading SNP, rs61067594 (*p* = 8.35 × 10^−11^), on chromosome 3, was found to be encoded by the *GMNC* gene and is strongly associated with white matter brain age gaps [32]. We also identified other genes, *SNAR-I* and *OSTN*. *OSTN* is induced by membrane depolarization in human brain neurons and restricts activity-dependent dendritic growth [47], a crucial step in brain development that is impacted by aging. Interestingly, the *SLC25A20* gene, part of the mitochondrial solute carrier family (SLC25), serves as a marker of mitochondrial dysfunction in the brain, which accompanies the development of Alzheimer’s disease, a disorder linked to brain aging [48,49].

We then calculated the PRS using 222,849 SNPs based on a more conservative threshold of *P_T_* = 0.05 (see Table S4 and Figure S2), which explains 76.8% of the variation in BAGs. The PRS was a significant predictor of the BAG in the multivariate model (Table S2). However, none of the PRS × exposure interactions were found to be significant (i.e., absence of G × E interaction effects; results not shown).

## 4. Discussion

In this study, we conducted both an XWAS and a GWAS on white matter brain aging within the UK Biobank cohort. We identified several significant environmental and lifestyle factors associated with the WM BAG. Key exposures such as smoking, specific dietary habits, sun exposure, and mobile use were found to influence brain aging. Genes highly related to brain function, structure, and development were found to be associated with the WM BAG. These findings highlight the critical role of the lifestyle/environment as well as genetic factors in brain health, although no significant G × E interactions were observed, suggesting that genetic predispositions may not strongly modify these environmental effects in this context.

Smoking was the most prominent factor associated with accelerated brain aging. Our study showed that current tobacco smoking was positively associated with the WM BAG, with the largest effect size. This result is consistent with the extensive evidence that smoking promotes oxidative stress and inflammation, key processes that accelerate neurodegeneration and cognitive decline [50,51,52]. The harmful effects of smoking on the brain are well-documented, with studies indicating that smoking leads to the production of reactive oxygen species that damage brain cells and accelerate aging-related processes, including the development of Alzheimer-like pathologies [4,53]. We have also previously demonstrated that smoking causes brain aging by using a robust Mendelian randomization method, reinforcing the need for targeted public health interventions [27].

Dietary habits also emerged as significant determinants of brain aging. For example, while the intake of oily fish and beef was associated with an increased WM BAG, suggesting an acceleration of brain aging, the cereal intake was linked to a reduction in the WM BAG, indicating potential protective effects. These findings align with research showing the neuroprotective benefits of certain nutrients, such as those found in cereals, which are rich in key nutrients, antioxidants, and fiber, contributing to better brain health [54,55,56]. Conversely, diets high in red meats, like beef, have been associated with higher levels of inflammation, potentially accelerating brain aging [57,58,59,60,61]. However, research that isolates the effects of single nutrients or foods often yields inconsistent findings and overlooks the broader context of dietary habits [62,63]. Therefore, future studies should prioritize examining comprehensive dietary patterns to gain a more accurate understanding of their influence on brain aging.

Unexpectedly, the use of UV protection was associated with an accelerated WM BAG. While UV protection is generally recommended to prevent photoaging, DNA damage, and photocarcinogenesis [64], this finding might reflect underlying behavioral patterns, such as higher overall sun exposure or unaccounted lifestyle factors. Individuals using UV protection might spend more time outdoors, increasing their exposure to environmental stressors despite using protective measures. Additionally, using solarium/sunlamps was also linked to accelerated brain aging in our study, potentially due to prolonged UV exposure leading to oxidative stress and neuroinflammation [65,66]. Further research is needed to fully understand the relationship between UV exposure, protection practices, and brain aging, considering factors like an outdoor occupation and seasonal variations.

Additionally, our study found that increased mobile phone use was associated with a decrease in the WM BAG, suggesting a protective effect against brain aging. This finding is intriguing and may reflect the cognitive engagement that mobile phone use can provide, such as through social interaction, information retrieval, and various cognitive activities that could contribute to maintaining cognitive function [67,68,69]. However, more research is needed to confirm this association and to explore the underlying mechanisms.

In addition to nongenetic factors, we also identified several important genetic risk factors of brain aging in GWAS: the *IP6K1* gene encodes a kinase that plays key roles in brain insula function [46], the *GMNC* gene encodes a protein in cellular processes critical to the development of white matter tracts [32], the *OSTN* gene restricts activity-dependent dendritic growth as a crucial step in brain development [47], and the *SLC25A20* gene has served as a marker of mitochondrial dysfunction in the brain, associated with Alzheimer’s disease [48,49]. Despite identifying several significant genetic and environmental factors affecting the WM BAG, we did not detect any significant G × E interactions. This suggests that the environmental factors studied have a relatively consistent impact on brain aging across different genetic backgrounds. The absence of interaction effects may indicate that these environmental exposures influence brain aging independently of genetic susceptibility, or it could reflect the need for more sophisticated analytical methods or larger sample sizes to detect subtle interactions.

This study’s primary strength lies in its comprehensive, multivariate approach using XWAS, allowing for a broad examination of multiple environmental factors without prior hypotheses. This method provides a nuanced understanding of the multifactorial nature of brain aging. Moreover, this study is among the first to explore such a wide array of environmental variables in relation to brain aging while also considering genetic susceptibility.

However, there are limitations to this study. Firstly, it includes potential selection bias due to the relatively healthy volunteers of the UK Biobank population, which may not fully represent the general population [70]. Secondly, reliance on self-reported data collected at baseline for the environmental factors, including dietary habits, could introduce recall or misclassification bias. Additionally, most dietary variables captured in the UK Biobank reflect short-term dietary practices rather than long-term habits, which may limit our ability to establish causality between the diet and brain aging, and future studies with repeated dietary assessments are needed to confirm these findings. As an exploratory analysis, the findings should be interpreted with caution, and the cross-sectional design limits the ability to establish causality. Last, the absence of observed G × E interactions might indicate either the uniform impact of these exposures or the need for more sophisticated models to detect subtle effects.

The findings from this study highlight several modifiable factors, such as smoking, dietary habits, UV protection use, and mobile phone use, which can be addressed in clinical settings to reduce brain aging and cognitive decline. Clinicians can guide individuals toward more holistic lifestyle changes, such as balanced UV exposure, healthier dietary choices, and responsible mobile phone use, to enhance brain health. From a public health perspective, interventions like smoking cessation programs and dietary modifications can help mitigate brain aging across populations. Although no significant G × E interactions were observed, the relatively consistent impact of these environmental factors suggests the broad applicability of these strategies. Given the exploratory nature of the findings, future confirmatory studies are warranted to validate these associations and refine recommendations, ensuring more personalized and effective interventions.

## Figures and Tables

**Table 1:** Descriptive statistics of the cohort used in this study.

Characteristic	Group	Results
N		30,375
Age (mean (SD))		55.47 (7.44)
Sex (n (%))	Male	14,704 (48.41)
	Female	15,671 (51.59)
BMI (mean (SD))		26.75 (4.25)
Education (n (%))	College or university degree	13,483 (44.39)
	A-levels/AS-levels or equivalent	3889 (12.80)
	O-levels/GCSEs or equivalent	5929 (19.52)
	CSEs or equivalent	1235 (4.07)
	NVQ, HND, HNC, or equivalent	1726 (5.68)
	Other professional qualifications, e.g., nursing, teaching	1531 (5.04)
Townsend deprivation index (mean (SD))		−1.86 (2.73)
Household income (n (%))	Less than 18,000	3293 (10.84)
	18,000 to 30,999	6227 (20.50)
	31,000 to 51,999	8300 (27.33)
	52,000 to 100,000	7625 (25.10)
	Greater than 100,000	2007 (6.61)
WM BAG (mean (SD))		0.14 (3.45)

**Table 2:** Associations of BAGs with the 17 variables selected in the univariate analysis of XWAS. * *p* < 0.05, ** *p* < 0.01, *** *p* < 0.001.

Category	Field ID	Description	Data Type	β (LB, UB)	*p*-Value	BH-Adjusted *p*-Value
Electronic device use	1110	Length of mobile phone use	Ordinal	−0.05 (−0.08, −0.02)	6.00 × 10^−04^	3.00 × 10^−3^ ***
Smoking	20160	Ever smoked	Binary	0.21 (0.13, 0.29)	8.40 × 10^−8^	1.00 × 10^−6^ ***
Smoking	20116	Smoking status (never vs. previous, current)	Binary	0.29 (0.22, 0.36)	1.23 × 10^−14^	1.97 × 10^−13^ ***
Smoking	1239	Current tobacco smoking	Binary	0.63 (0.49, 0.77)	<2 × 10^−16^	3.4 × 10^−15^ ***
Smoking	1249	Past tobacco smoking	Binary	0.14 (0.07, 0.23)	3.00 × 10^−4^	2.00 × 10^−3^ ***
Diet	1329	Oily fish intake	Ordinal	0.08 (0.03, 0.12)	3.00 × 10^−4^	2.00 × 10^−3^ ***
Diet	1369	Beef intake	Ordinal	0.10 (0.05, 0.14)	3.12 × 10^−5^	3.00 × 10^−4^ ***
Diet	1379	Lamb/mutton intake	Ordinal	0.06 (0.002, 0.11)	4.00 × 10^−2^	4.00 × 10^−2^ *
Diet	6144	Eat eggs, dairy, wheat, sugar (all vs. some)	Binary	0.27 (0.18, 0.37)	1.34 × 10^−8^	2.00 × 10^−7^ ***
Diet	1408	Cheese intake	Ordinal	−0.04 (−0.07, −0.004)	0.03	4.00 × 10^−2^ *
Diet	1428	Spread type used (alternative margarine vs. other types)	Binary	0.14 (0.06, 0.21)	3.00 × 10^−4^	2.00 × 10^−3^ ***
Diet	1458	Cereal intake	Numerical	−0.05 (−0.07, −0.04)	1.58 × 10^−13^	2.37 × 10^−12^ ***
Diet	1498	Coffee intake	Numerical	0.06 (0.04, 0.07)	8.41 × 10^−9^	1.00 × 10^−7^ ***
Diet	1528	Water intake	Numerical	0.03 (0.01, 0.05)	7.00 × 10^−4^	3.00 × 10^−3^ ***
Diet	1538	No dietary changes in the last 5 years	Binary	0.13 (0.05, 0.20)	1.00 × 10^−3^	6.00 × 10^−3^ **
Sun exposure	2267	Use of sun/UV protection most of the time (most of the time vs. other)	Binary	0.20 (0.12, 0.27)	3.41 × 10^−7^	4.00 × 10^−6^ ***
Sun exposure	2277	Frequency of solarium/sunlamp use	Numerical	0.01 (0.003, 0.02)	7.00 × 10^−3^	2.00 × 10^−2^ **

## Data Availability

The raw genetic and phenotypic data used for this study can be found in the UK Biobank (http://www.ukbiobank.ac.uk/) (accessed on 1 July 2024). The UKB application number is 74376.

## Supplementary Materials

The following supporting information can be downloaded at: https://www.mdpi.com/article/10.3390/genes15101285/s1, Figure S1. (A) Predicted brain age vs. chronological without age bias correction. (B) Predicted brain age vs. chronological with age bias correction. Mean Absolute Error (MAE): MAE is a measure of the average absolute difference between the predicted values and the actual values. It quantifies the magnitude of errors without considering their direction (positive or negative). Figure S2. Bar plot showing results at a set of GWAS P-value thresholds for PRS calculation on BAG. Table S1. A list of 39 regional white matter (WM) integrity measured by fractional antitropy. Table S2. The final selected 11 exposure variables from multivariate analysis of XWAS. * *p* < 0.05, ** *p* < 0.01, *** *p* < 0.001. Table S3. Annotation of 185 significant SNPs (*p*-value < 1 × 10^−6^) from GWAS analysis. Table S4. Polygenic risk score results for a set of GWAS *p*-value thresholds.
