# Supplementary material for: Nongenetic and Genetic Factors Associated with White Matter Brain Aging: Exposome-Wide and Genome-Wide Association Study
Source: Genes (Basel). 2024 Sep 30;15(10):1285. doi: 10.3390/genes15101285 (PMC11507416; doi:10.3390/genes15101285)
Supplement: Supplementary file 1 [file genes-15-01285-s001.zip › genes-3225986-supplementary.pdf]

Nongenetic and genetic factors associated with white matter brain aging: exposome-wide and genome-wide association study

Supplement

Figures and Tables

Figure S1. (A) Predicted brain age vs. chronological without age bias correction. (B) Predicted brain age vs. chronological with age bias correction. Mean Absolute Error (MAE): MAE is a measure of the average absolute difference between the predicted values and the actual values. It quantifies the magnitude of errors without considering their direction (positive or negative).

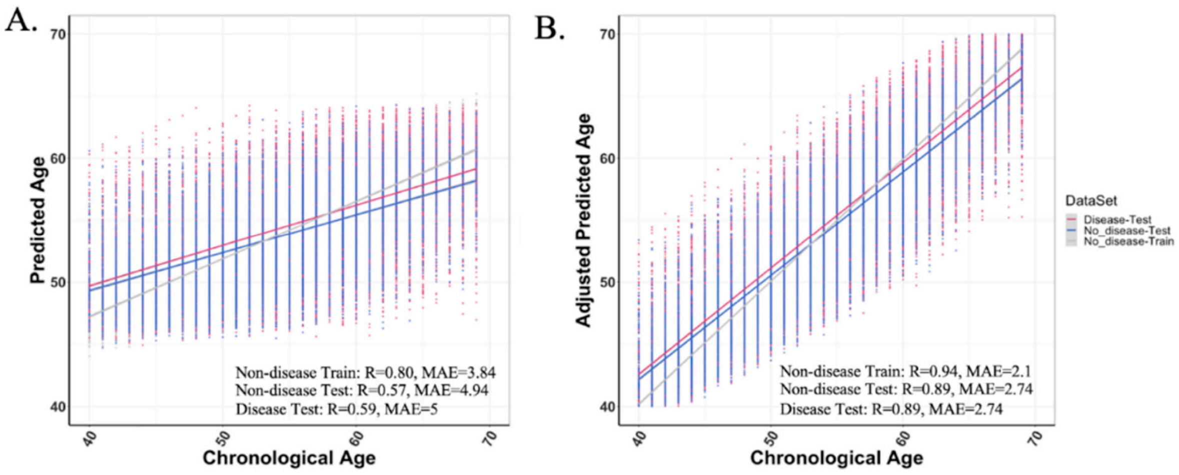

Figure S2. Bar plot showing results at a set of GWAS P-value thresholds for PRS calculation on BAG.

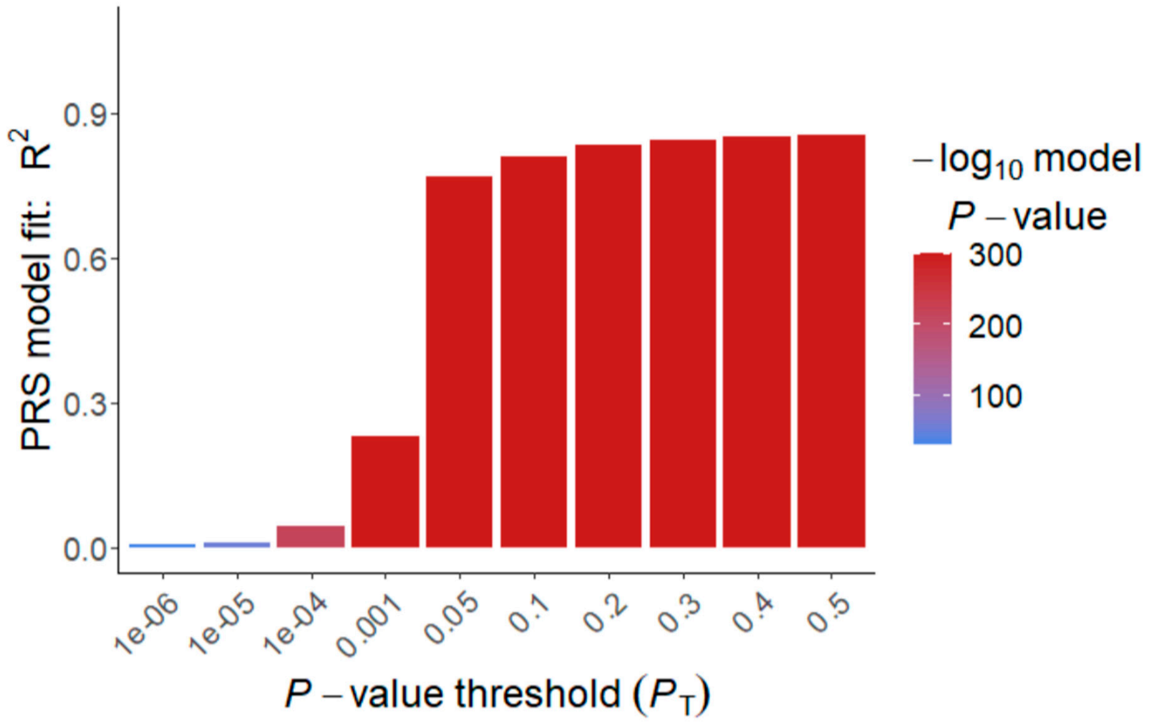

Table S1. A list of 39 regional white matter (WM) integrity measured by fractional antitropy.

**Region of Interest**	**Abbreviations**	**Hemisphere**
Inferior cerebellar peduncle	ICP	/
Genu of corpus callosum	GCC *	/
Body of corpus callosum	BCC *	/
Splenium of corpus callosum	SCC *	/
Fornix	FX *	/
Corticospinal tract	CST-L	Left
Corticospinal tract	CST-R	Right
Anterior limb of internal capsule	ALIC-L *	Left
Anterior limb of internal capsule	ALIC-R	Right
Posterior limb of internal capsule	PLIC-L *	Left
Posterior limb of internal capsule	PLIC-R	Right
Retrolenticular part of internal capsule	RLIC-L	Left
Retrolenticular part of internal capsule	RLIC-R	Right
Anterior corona radiata	ACR-L *	Left
Anterior corona radiata	ACR-R *	Right
Superior corona radiata	SCR-L	Left
Superior corona radiata	SCR-R	Right
Posterior corona radiata	PCR-L	Left
Posterior corona radiata	PCR-R *	Right
Posterior thalamic radiation	PTR-L *	Left
Posterior thalamic radiation	PTR-R *	Right
Sagittal stratum	SS-L	Left
Sagittal stratum	SS-R	Right
External capsule	EX-L *	Left
External capsule	EX-R *	Right
Cingulum cingulate gyrus	CGC-L	Left
Cingulum cingulate gyrus	CGC-R	Right
Cingulum hippocampus	CHG-L	Left
Cingulum hippocampus	CHG-R	Right
Fornix (cres)/stria terminalis	FXST-L *	Left
Fornix (cres)/stria terminalis	FXST-R *	Right
Superior longitudinal fasciculus	SLF-L	Left
Superior longitudinal fasciculus	SLF-R	Right
Superior fronto-occipital fasciculus	SFO-L *	Left
Superior fronto-occipital fasciculus	SFO-R	Right
Uncinate fasciculus	UN-L	Left
Uncinate fasciculus	UN-R	Right

Tapetum	TAP-L	Left
Tapetum	TAP-R	Right

* indicates 16 fractional antitropy measures were chosen in the random forest machine learning model from the training data set to predict the WM brain age in the testing data set

Table S2. The final selected 11 exposure variables from multivariate analysis of XWAS. * p<0.05, ** p<0.01, *** p<0.001

Category	Covariate Name	Beta (LB , UB)	P-value
	Age	-0.13 (-0.13 , -0.12)	< 2E-16 ***
	Sex (male)	0.95 (0.88 , 1.01)	< 2E-16 ***
	BMI	0.02 (0.009 , 0.02)	1.97E-05 ***
Electronic Device Use	Length of mobile phone use	-0.04 (-0.06 , -0.01)	0.005 **
Smoking	Current tobacco smoking	0.08 (-0.04 , 0.20)	0.17
Diet	Oily fish intake	0.04 (0.005 , 0.08)	0.02 *
Diet	Beef intake	0.01 (-0.03 , 0.05)	0.6
Diet	Lamb/mutton intake	0.02 (-0.03 , 0.07)	0.45
Diet	Cereal intake	-0.03 (-0.07 , -0.004)	0.03 *
Diet	Coffee intake	0.04 (0.004 , 0.07)	0.03 *
Diet	Water intake	-0.009 (-0.04 , 0.02)	0.58
Diet	No Dietary changes in the last 5 years	0.02(-0.05 , 0.08)	0.57
Sun Exposure	Use of sun/uv protection most of the time (Most of the time vs other)	0.07 (0.01 , 0.14)	0.02 *
Sun Exposure	Frequency of solarium/sunlamp use	0.02 (-0.02 , 0.05)	0.32
	PRS	2.73 (2.70 , 2.76)	< 2E-16 ***

Table S3. Annotation of 185 significant SNPs (p-value <1e-6) from GWAS analysis.

Chr	SNP	BP	Ref	Alt	Func.refGene	Gene.refGene
3	rs142003765	39479611	A	G	intergenic	RPSA;MOBP
3	rs73080312	48737847	C	T	intronic	IP6K2
3	rs11715689	48770281	C	T	intergenic	IP6K2;PRKAR2A
3	rs11715713	48770306	C	T	intergenic	IP6K2;PRKAR2A
3	rs11715835	48770732	C	T	intergenic	IP6K2;PRKAR2A
3	rs13084000	48772714	G	C	intergenic	IP6K2;PRKAR2A
3	rs13074318	48816725	C	T	intronic	PRKAR2A
3	rs34037363	48821036	C	T	intronic	PRKAR2A
3	rs73080361	48832146	G	A	intronic	PRKAR2A
3	rs11713694	48833615	G	T	intronic	PRKAR2A
3	rs13094900	48837732	G	A	intronic	PRKAR2A
3	rs34930159	48850838	-	A	intronic	PRKAR2A
3	rs11709246	48859498	G	A	intronic	PRKAR2A
3	rs71324924	48865444	G	A	intronic	PRKAR2A
3	rs67332931	48866548	C	T	intronic	PRKAR2A
3	rs55761431	48885212	G	A	UTR5	PRKAR2A
3	rs11709092	48887027	A	C	ncRNA_exonic	PRKAR2A-AS1
3	rs11708022	48893136	G	A	intergenic	PRKAR2A-AS1;SLC25A20
3	rs112837572	48901171	C	T	intronic	SLC25A20
3	rs13073997	48911647	C	T	intronic	SLC25A20
3	rs67060340	48915321	C	T	intronic	SLC25A20
3	rs398105856	48920995	-	A	intronic	SLC25A20
3	rs34477108	48941782	A	G	intergenic	SLC25A20;ARIH2OS
3	rs116432667	48947589	C	T	intergenic	SLC25A20;ARIH2OS
3	rs80170003	48951640	T	C	intergenic	SLC25A20;ARIH2OS
3	rs112736626	48952483	G	A	intergenic	SLC25A20;ARIH2OS
3	rs56318350	48953777	G	A	intergenic	SLC25A20;ARIH2OS
3	rs13087930	48961726	A	G	intronic	ARIH2
3	rs34509487	48964108	C	T	intronic	ARIH2
3	rs11717978	48969036	T	A	intronic	ARIH2
3	rs11719457	48974385	C	T	intronic	ARIH2
3	rs111661834	48977202	A	C	intronic	ARIH2
3	rs13090839	48977633	C	T	intronic	ARIH2
3	rs13086240	49001409	G	A	intronic	ARIH2
3	rs11716614	49017208	C	G	intronic	ARIH2
3	rs34504524	49032381	C	T	intronic	P4HTM

3	rs71324929	49044713	C	T	upstream;downstream	WDR6;P4HTM
3	rs3923475	49055788	G	T	intronic	DALRD3
3	rs11706052	49064110	A	G	intronic	IMPDH2
3	rs61729488	49067904	T	C	exonic	QRICH1
3	rs13080725	49076092	G	A	intronic	QRICH1
3	rs13072554	49087211	G	A	intronic	QRICH1
3	rs35108630	49088356	T	A	intronic	QRICH1
3	rs35102816	49109585	G	A	intronic	QRICH1
3	rs11718982	49132554	C	T	upstream;downstream	QRICH1;QARS1
3	rs34326553	49134429	A	G	intronic	QARS1
3	rs35673421	49139406	G	A	intronic	QARS1
3	rs5030795	49141116	G	A	exonic	QARS1
3	rs11552724	49156473	C	G	exonic	USP19
3	rs10154895	49174356	G	T	intergenic	LAMB2;LAMB2P1
3	rs9880088	49178990	G	A	intergenic	LAMB2;LAMB2P1
3	rs55921200	49189316	T	C	downstream	LAMB2P1
3	rs9862483	49195441	G	A	intergenic	LAMB2P1;CCDC71
3	rs9813813	49207093	T	A	intergenic	CCDC71;KLHDC8B
3	rs9814987	49207849	T	C	intergenic	CCDC71;KLHDC8B
3	rs34571182	49209057	T	A	UTR5	KLHDC8B
3	rs13096406	49209058	C	A	UTR5	KLHDC8B
3	rs11706189	49210833	C	A	intronic	KLHDC8B
3	rs11713297	49213030	T	C	intronic	KLHDC8B
3	rs9837237	49215583	G	T	intronic	C3orf84
3	rs9837625	49215966	C	T	exonic	C3orf84
3	rs9835439	49220330	G	C	intronic	C3orf84
3	rs66991555	49228643	A	G	intronic	C3orf84
3	rs9855123	49229457	C	T	upstream	C3orf84
3	rs74477111	49233006	G	A	intergenic	C3orf84;IHO1
3	rs36095141	49234113	-	T	intergenic	C3orf84;IHO1
3	rs113943883	49238722	G	A	intronic	IHO1
3	rs34566463	49245077	T	C	intronic	IHO1
3	rs11719220	49245201	A	G	intronic	IHO1
3	rs35852153	49247462	C	T	intronic	IHO1
3	rs71324939	49248608	T	C	intronic	IHO1
3	rs11710247	49249800	G	T	intronic	IHO1
3	rs112890859	49252583	A	G	intronic	IHO1
3	rs147876732	49254385	C	T	intronic	IHO1
3	rs35060561	49255503	T	C	intronic	IHO1

3	rs9826525	49264615	T	A	intronic	IHO1
3	rs67286839	49265744	C	T	intronic	IHO1
3	rs11720460	49269270	C	A	intronic	IHO1
3	rs73084135	49272218	A	G	intronic	IHO1
3	rs113186424	49272726	T	C	intronic	IHO1
3	rs34784192	49274823	G	T	intronic	IHO1
3	rs35185152	49277521	C	T	intronic	IHO1
3	rs9850917	49286983	A	G	intronic	IHO1
3	rs111256226	49288614	T	C	intronic	IHO1
3	rs9847839	49289003	T	C	intronic	IHO1
3	rs9828310	49289387	C	G	intronic	IHO1
3	rs9990153	49291883	C	T	intronic	IHO1
3	rs13068038	49294250	C	A	exonic	IHO1
3	rs12715434	49295025	G	C	UTR3	IHO1
3	rs9880309	49304329	G	C	intergenic	IHO1;C3orf62
3	rs34343820	49309534	C	G	intronic	C3orf62
3	rs10049462	49309684	C	T	intronic	C3orf62
3	rs9311439	49310408	A	G	intronic	C3orf62
3	rs530323357	49312952	-	TTTTTTT	intronic	C3orf62
3	rs67831908	49313513	T	G	intronic	C3orf62
3	rs13077498	49313978	C	T	exonic	C3orf62
3	rs56306491	49314960	G	A	UTR3	USP4
3	rs9870030	49332709	G	A	intronic	USP4
3	rs112274965	49342417	G	A	intronic	USP4
3	rs9844757	49343601	T	G	intronic	USP4
3	rs35446411	49350033	A	C	exonic	USP4
3	rs9864406	49352619	T	C	intronic	USP4
3	rs34915642	49358646	G	A	intronic	USP4
3	rs35869135	49359102	A	G	intronic	USP4
3	rs9873994	49359943	T	C	intronic	USP4
3	rs9863142	49366741	G	C	intronic	USP4
3	rs34514189	49373045	A	G	intronic	USP4
3	rs34567776	49375349	C	G	intronic	USP4
3	rs67882627	49380441	A	T	intergenic	USP4;GPX1
3	rs9818758	49382925	G	A	intergenic	USP4;GPX1
3	rs11716445	49406095	G	A	intronic	RHOA
3	rs112837936	49466021	G	A	intronic	NICN1
3	rs111353936	49475898	G	A	intergenic	NICN1;DAG1
3	rs73073015	49503166	G	A	intergenic	NICN1;DAG1

3	.	49526044	T	A	intronic	DAG1
3	rs113176147	49534439	G	T	intronic	DAG1
3	rs55873331	49597055	C	T	intronic	BSN
3	rs56116382	49606188	A	C	intronic	BSN
3	rs138320228	49626600	C	T	intronic	BSN
3	rs73074866	49626666	C	T	intronic	BSN
3	rs11716159	49631585	G	A	intronic	BSN
3	rs11710905	49651534	C	T	intronic	BSN
3	rs12185979	49651970	C	G	intronic	BSN
3	rs3197999	49721532	G	A	exonic	MST1
3	rs55662945	49732699	C	T	intronic	RNF123
3	rs34823813	49749976	G	A	exonic	RNF123
3	rs9853352	49813258	A	G	intronic	IP6K1
3	rs112101327	49813826	T	C	intronic	IP6K1
3	rs73077200	49826987	C	T	intergenic	IP6K1;CDHR4
3	rs73079004	49833904	G	C	intronic	CDHR4
3	rs73079018	49875327	T	C	intronic	TRAIP
3	rs9858428	49877585	G	T	intronic	TRAIP
3	rs200481589	49902948	T	A	intronic	CAMKV
3	rs11716961	49908338	G	A	upstream	CAMKV
3	rs73079033	49979174	T	C	intronic	RBM6
3	rs11717829	49979727	C	T	intronic	RBM6
3	rs111742464	50001393	G	T	intronic	RBM6
3	rs4688759	50008118	T	C	intronic	RBM6
3	rs11711814	50044813	T	G	intronic	RBM6
3	rs2624818	50056265	G	A	intronic	RBM6
3	rs2023953	50103917	G	A	exonic	RBM6
3	rs3216783	50144667	-	T	intronic	RBM5
3	rs73080976	50187326	G	A	ncRNA_intronic	SEMA3F-AS1
3	rs11710277	50199747	A	G	intronic	SEMA3F
3	rs146550622	190638421	T	C	intergenic	SNAR-I;OSTN
3	rs113180518	190638818	G	T	intergenic	SNAR-I;OSTN
3	rs112522420	190638851	C	T	intergenic	SNAR-I;OSTN
3	rs4624605	190640121	G	T	intergenic	SNAR-I;OSTN
3	rs4308324	190640134	A	T	intergenic	SNAR-I;OSTN
3	rs4308325	190640155	A	C	intergenic	SNAR-I;OSTN
3	rs73193907	190640357	G	T	intergenic	SNAR-I;OSTN
3	rs34886272	190641719	A	C	intergenic	SNAR-I;OSTN
3	rs61494018	190642391	A	G	intergenic	SNAR-I;OSTN

3	rs60386222	190643193	G	T	intergenic	SNAR-I;OSTN
3	rs61067594	190650833	G	A	intergenic	SNAR-I;OSTN
3	rs2363038	190656393	G	T	intergenic	SNAR-I;OSTN
3	rs13100545	190657895	A	G	intergenic	SNAR-I;OSTN
3	rs1159211	190666643	C	T	intergenic	SNAR-I;OSTN
3	rs4686570	190667970	G	A	intergenic	SNAR-I;OSTN
3	rs138133138	190668850	CATCCATGTAGTC	-	intergenic	SNAR-I;OSTN
3	rs9877502	190669518	G	A	intergenic	SNAR-I;OSTN
3	rs147182970	190670179	-	CAG	intergenic	SNAR-I;OSTN
3	rs200690866	190670370	T	C	intergenic	SNAR-I;OSTN
3	rs35327527	190670795	C	T	intergenic	SNAR-I;OSTN
5	rs11742770	64792619	G	A	intergenic	ADAMTS6;CENPK
5	rs72762414	64795432	C	T	intergenic	ADAMTS6;CENPK
5	rs201481959	64798938	T	-	intergenic	ADAMTS6;CENPK
5	rs10940039	64804699	G	A	intergenic	ADAMTS6;CENPK
5	rs12517057	64805104	A	G	intergenic	ADAMTS6;CENPK
5	rs2166382	64805378	C	T	intergenic	ADAMTS6;CENPK
5	rs113394445	64806423	G	A	intergenic	ADAMTS6;CENPK
5	rs72762422	64806638	C	T	intergenic	ADAMTS6;CENPK
5	rs2367535	64807003	T	G	intergenic	ADAMTS6;CENPK
5	rs12523328	64811832	C	T	downstream	CENPK
5	rs11747574	64820922	A	C	intronic	CENPK
5	rs11741071	64821089	G	A	intronic	CENPK
5	rs72762426	64821563	A	G	intronic	CENPK
5	rs80347054	64823406	T	A	intronic	CENPK
5	rs113470058	64825789	G	A	intronic	CENPK
5	rs72762430	64831680	T	C	intronic	CENPK
5	rs75909203	64833621	T	C	intronic	CENPK
5	rs72762432	64833725	G	T	intronic	CENPK
5	rs72762433	64835134	T	C	intronic	CENPK
5	rs112535439	64846897	C	T	intronic	CENPK
5	rs3213798	64976687	C	T	intronic	SGTB

Table S4. Polygenic risk score results for a set of GWAS p-value thresholds.

Threshold	Fullmodel.R^2	Nullmodel.R^2	PRS.R^2	PRS.BETA	PRS.SE	PRS.P	Number of SNPs
0.000001	0.0347	0.0288	0.0059	10.0180	0.8716	1.73E-30	162
0.00001	0.0399	0.0288	0.0111	21.3449	1.3494	4.77E-56	328
0.0001	0.0719	0.0288	0.0432	111.7572	3.5165	1.06E-216	1253
0.001	0.2594	0.0288	0.2307	800.9563	9.7307	0	7039
0.05	0.7967	0.0288	0.7680	6058.5256	21.1324	0	222849
0.1	0.8367	0.0288	0.8080	8092.7961	24.6628	0	422884
0.2	0.8633	0.0288	0.8345	11246.2688	30.8647	0	802119
0.3	0.8743	0.0288	0.8456	14148.8855	36.9819	0	1172811
0.4	0.8797	0.0288	0.8509	17020.6133	43.3940	0	1538386
0.5	0.8824	0.0288	0.8536	19919.5687	50.1284	0	1897887
